# Unmet care needs in the oldest old with social loss experiences: results of a representative survey

**DOI:** 10.1186/s12877-020-01822-2

**Published:** 2020-10-20

**Authors:** Janine Stein, Margrit Löbner, Alexander Pabst, Hans-Helmut König, Steffi G. Riedel-Heller

**Affiliations:** 1 Institute of Social Medicine, Occupational Health and Public Health, Medical Faculty, University of Leipzig, Leipzig, Germany; 2 Department of Health Economics and Health Services Research, University Medical Center Hamburg-Eppendorf, Hamburg, Germany

**Keywords:** Loss experiences, Bereavement, Need assessment, Old age, Health services research

## Abstract

**Background:**

Loss experiences such as the loss of a spouse, a close relative or significant others become more likely in old age and may be strongly related to specific unmet health care needs. These unmet needs may often remain undetected and undertreated followed by a negative impact on well-being and social role functioning. The present study aims at exploring the relationship between loss experiences and specific unmet care needs in old age.

**Methods:**

As part of the study „Need assessment in the oldest old: application, psychometric examination and establishment of the German version of the Camberwell Assessment of Need for the Elderly (CANE)”, the adapted German version of the CANE was used in a population-representative telephone survey in a sample of 988 individuals aged 75+ years. Loss experiences within the last 12 months were assessed within the structured telephone survey. Descriptive and interferential statistical analyses were run in order to examine the association between loss experiences and occurring unmet care needs.

**Results:**

Overall, 29.7% of the oldest old reported at least one social loss with other relatives losses being the most frequent (12.5%), followed by non-family losses (10.7%). A significant relationship between loss experiences and a higher number of unmet care needs was observed, especially for close family losses. Other risk factors for unmet care needs were age, marital status, depression, social support and morbidity.

**Conclusions:**

This study provides, for the first time in Germany, data on the association between loss experiences and unmet needs. These findings may substantially contribute to the development of loss-specific interventions, effective treatment and health care planning for the bereaved elderly.

## Background

Bereavement and loss experiences, such as the loss of the spouse, close relatives, friends or significant others are common phenomena in the elderly [1]. Such loss experiences are not only more likely with age, but also require psychological re-adjustment from relatives and survivors, and can be associated with substantial health and psychosocial impairments [2–5]. Such effects of loss experiences and bereavement on health and other outcomes range from negative changes in routine health behaviors including physical activity, nutrition, sleep quality, alcohol consumption, tobacco use, and body weight status [6], reduced life satisfaction [7] to negative effects on patterns of health care utilization [8]. Moreover, a recent study showed that bereavement after spousal loss can negatively influence the quality of health care that individuals receive because the crisis caused by spousal death may negatively affect individuals’ abilities to maintain contact with health care providers [9]. Against this background, loss experiences and grief can be accompanied by unmet care needs in old age, that often remain undetected and can negatively affect well-being and role functioning [4]. The definition of care needs is based on the “capacity-to-benefit-concept“, covering the ability of individuals to benefit from healthcare provision. According to this concept, care needs are assumed to exist if there is potential for an effective treatment or health gain. Based on this concept, the Camberwell Assessment of Need in the Elderly (CANE) was developed in order to systematically assess the met and unmet care needs in older individuals [10]. Since its development, the CANE was translated into many languages and internationally used in a broad range of settings. Correspondingly, care needs are met if they receive appropriate support or assistance. On the other hand, unmet care needs exist, if there is currently no adequate intervention for it, the wrong type or the wrong level of help.

According to the National Health & Aging Trends Study (NHATS), a nationally representative survey of Americans aged 65 or older, the vast majority of older adults receives help by at least one informal caregiver [11]. Family caregivers such as spouses or other family members play an important role in this context [12], as they provide help with basic activities of daily living (ADL) and instrumental activities of daily living (IADL). Thus, they present important resources in supporting elderly care recipients [13]. Current research has investigated family caregiver factors associated with unmet needs of older adults [14], finding associations e.g. with younger age of caregivers, the type of family relationship (sons), the living situation (apart from care recipient), or experiencing high levels of burden. On the other hand, little is known about the situation of elderly people in case a potential informal caregiver passes away. Consequently, the relationship between loss experiences and specific, associated unmet needs in old age is widely underresearched. Data for Germany are widely missing. Particularly in view of the demographic change in Germany, but also in other European Countries, gaining knowledge about this relationship may have important practical implications for providing adequate and appropriate care for the oldest old. Thus, the purpose of this study was to examine the frequency and distribution of loss experiences and their association with unmet needs in the elderly aged 75+ years. Therefore the following research questions are addressed:

1. How frequent is loss experience within individuals aged 75 years and older and what types of social loss experience exist in this age group?
2. How frequent are unmet needs in individuals aged 75 years and older with loss experience compared to individuals without loss?
3. How is loss experience associated with unmet needs in the elderly? What other factors play a role in this context?

## Methods

### Study design

This cross-sectional study collected data from July 2016 to October 2016 by applying a representative telephone survey (General Population Survey, GPS, 75+ years). The telephone survey was implemented by USUMA, a leading market, opinion and social research institute in Germany as part of the project “Needs assessment in the oldest old: application, psychometric examination and establishment of the adapted German version of the Camberwell Assessment of Need for the Elderly (CANE)” funded by the German Research Foundation (DFG). The aims of this project were to investigate the met and unmet health care needs of the oldest old (75+ years) based on the adapted German version of the CANE that was developed within the preceding pilot project [15, 16]. In the survey, standardized structured computer-assisted telephone interviews (CATI) were carried out. Prior to the main survey a pretest was conducted in order to test and to adjust the interview. All five interviewers were trained by members of the study team.

### Survey procedures

The selection of households and study participants was based on the sampling system of the Association of German Market and Social Research Agency (ADM) that includes registered and non-registered telephone numbers. In order to ensure representativeness of the study sample to the German population aged 75 years and older, landline numbers from the whole of Germany were randomly selected proportional to the population structure and regionally stratified by federal state and community size. The target person (75+ years) within the selected household was randomly selected following the Kish selection grid method [17]. In this process, all persons relevant to the target group (at least 75 years old) were first identified via a contact person. In the second step, the Kish grid randomly and independently from the interviewer selected the person to be interviewed. By using this selection method, the equal probability of participation for each member in the age group “75 years or older” living in the selected household and representativeness of the sample for the German population aged 75+ years was ensured. The initial criteria for inclusion in the telephone survey were: 1) being 75 years and older, 2) sufficient German language skills as well as speech comprehension, and 3) sufficient hearing ability. Excluded from participation in the telephone survey were individuals with 1) insufficient hearing, speaking and speech comprehension, and 2) comprehension problems due to cognitive impairment or suspected dementia (determined via the 6-item cognitive impairment test, 6-CIT [18, 19]) including the inability to give informed consent to the study.

### Sample

**Fig. 1:**
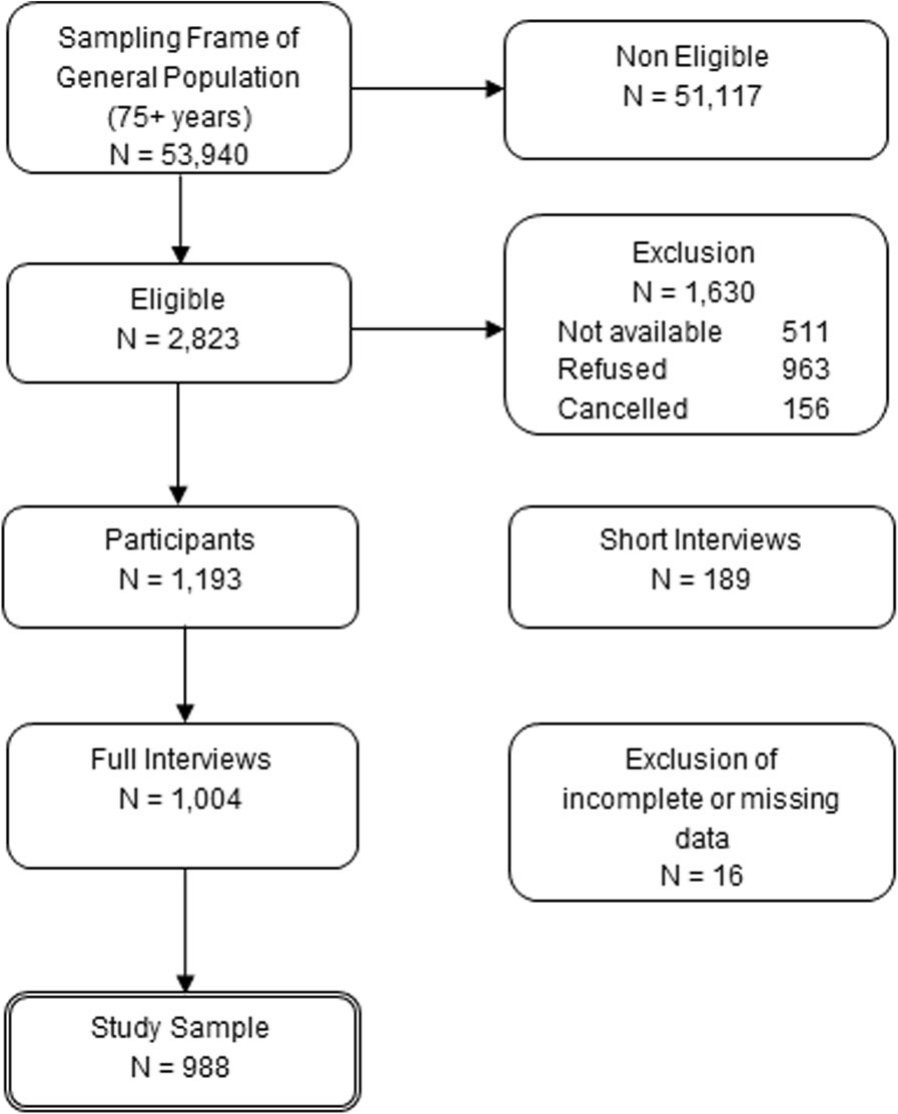
Flow chart of sample selection in this study

A total of 53,940 people were randomly selected from which 51,117 were non-eligible. Reasons were wrong telephone number (*N* = 30,279), occupied/not reached (*N* = 5366), no time or interest (*N* = 2582), person of the target group was not present or available (*N* = 164), insufficient German language skills (*N* = 234), telephone connection not belonging to target group (company, association) (*N* = 664), no person of the target group (aged 75+) living in household (*N* = 11,397), cognitive difficulties or deafness (*N* = 431). In total, 2823 persons were contacted by telephone, of which 511 persons (18.1%) were not available, 963 persons (34.1%) refused to participate and 156 persons (5.5%) cancelled the telephone conversation. Finally, a total of 1193 telephone interviews (1004 full interviews with an average duration of 40 min and 189 short interviews with an average duration of 7 min and 12 s) were conducted by USUMA, resulting in a response rate of 42.3%. The short interviews were discarded from the current study; they were carried out when the preceding 6-item cognitive impairment test (6-CIT) revealed a score of 7 or more points, indicating dementia. In addition, 16 individuals with incomplete or missing information on sociodemographic variables were excluded from the study sample. Ultimately, the analyses of the present work were based on a sample size of 988 persons. Figure 1 shows the sample selection process in detail.

### Social loss experiences

In the present study, social loss experiences refer to bereavement due to the death of a loved one. It does not include other bereavement experiences such as moving away, or hospitalization. Data regarding the frequency of social loss experience were collected by using a single item of the Leipziger Lebensereignis-Liste (LLL). The LLL was designed and adapted for the elderly on the basis of established instruments for the assessment of stressful live events such as the Recent Life Changes Questionnaire [20], Social Readjustment Rating Scale [21], and the Life Events and Difficulties Schedule [22]. The questionnaire consists of 10 items, each referring to another stressful life event. In the present study the loss-specific item of the LLL was used: Participants were asked whether a close relative or significant other on whose support they were dependent passed away within the last 12 months (1 “yes” vs. 0 “no”). If this question was affirmed the participants were additionally asked who died. Based on this information, a variable was built containing four types of losses: 0 “no loss”; 1 “close family loss” including the loss of a partner/spouse or child; 2 “other relatives loss” including the loss of siblings or other relatives; and 3 “non-family loss” including the loss of friends, neighbors or others. Participants who experienced more than one loss were ranked into the category that is likely to have the higher impact on unmet needs, assuming that the loss of close family members has the highest impact, and the loss of non-family members has the lowest impact.

### Camberwell assessment of need for the elderly (CANE)

The main instrument in the standardized structured computer-assisted telephone interviews was the German-language version of the Camberwell Assessment of Need for the Elderly (CANE) [16, 23]. The CANE was developed in the United Kingdom for the assessment of needs in the elderly covering environmental, social, physical and psychological met and unmet needs [10]. As developers of the German version the authors had the permission to use the instrument within the study. The CANE shows good psychometric properties [24]. A detailed description of the instrument can be found elsewhere [25]. CANE need sections were coded as either 0 (“no need” or “met need”) or 1 (“unmet need”). A sum score of the 25 CANE items was calculated, indicating the total number of care needs rated by the participants as unmet.

### Other instruments

Depressive symptoms were determined via the German 15-item version of the Geriatric Depression Scale (reliability coefficient r_tt_ = 0.90; internal consistency Cronbach alpha = .91) [26]. The scale is in the public domain (https://web.stanford.edu/~yesavage/GDS.html). The visual analogue scale (VAS) of the EQ-5D questionnaire, which shows high convergent validity (0.90–0.99), was used to assess participants´ health-related quality of life [27, 28]. The instrument is available in different languages (see https://euroqol.org/). The Lubben Social Network Scale (LSNS-6) was included in the interview in order to assess the social networks and support in the elderly participants [29]. The instrument is freely available in different length versions and various languages (see https://www.bc.edu/content/bc-web/schools/ssw/sites/lubben/description/versions-of-the-lsns.html). The LSNS-6 shows high levels of internal consistency, stable factor structures, and high correlations with criterion variables [30]. Moreover, socio-demographic data of participants were collected, including age, gender, domicile, marital status, and number of illnesses (morbidity). Educational level of participants (high, moderate, low) was determined via the International Standard Classification of Education (ISCED, [31]. For the GDS, EQ-5D VAS and LSNS sum scores were computed. GDS scores ranged from 0 to 15 with higher scores indicating more severe depressive symptoms. The EQ-5D VAS ranged from 0 to 100 with higher scores indicating higher health-related quality of life. LSNS-6 total score is an equally weighted sum of six items (scores ranging from 0 to 30) with a higher score indicating more social engagement.

### Statistical analyses

All analyses were performed using the programs IBM statistics SPSS version 24 for Windows (SPSS Inc., Chicago, IL) and Stata 13.1 SE (Stata-Corp LP, College Station, TX). Case numbers are presented as unweighted frequencies. All other data were weighted to represent the distribution of household size, age, gender and region in the German general population. For all computations, the significance level was set to α ≤ 0.05.

Descriptive results are presented as mean ± SD or number of cases with percentages, as appropriate. Differences between individuals with or without loss experiences and unmet needs were assessed via chi-square tests for nominal variables. Regression analyses were run in order to investigate the impact of social loss experiences and other risk factors (such as sex, age, marital status, education, domicile, depression, health-related quality of life, social engagement and number of illnesses) on the occurrence of unmet needs in the elderly. Traditional linear models were considered inappropriate because the distribution of unmet needs was substantially right-skewed. Instead, we performed a negative binomial regression model [32] to estimate the number of unmet needs from age, gender, marital status, education, domicile, depression, quality of life, social network, morbidity, and social loss experiences. This model was preferred over Poisson regression because of observed overdispersion, i.e. the variance of unmet needs is greater than its mean. The estimated coefficients of the negative binomial regression model were transformed to incidence-rate ratios (IRR), representing the percent change in the number of unmet needs associated with a 1-unit increase in the predictor.

## Results

### Characteristics of the study sample

Table 1 shows the socio-demographic characteristics of the study sample. On average, participants were 80.48 (SD = 4.70) years old. Approximately two thirds of the sample was female (59.4%). Educational level was moderate (41.5%) or high (49.2%) in most of the cases. Nearly half of the sample was widowed (45.8%) while 39.1% of the participants were married and 15.1% single or divorced. Most of the participants (56.1%) lived alone in a private household.

**Table 1:** Socio-demographic characteristics of the sample (*N* = 988)

Characteristics	
**Age (in years)**	
Mean (SD)	80.48 (4.70)
Range	75–99
**Gender, n (%)**	
Male	380 (40.6)
Female	608 (59.4)
**ISCED, n (%)**	
Low	93 (9.3)
Moderate	413 (41.5)
High	482 (49.2)
**Marital Status, n (%)**	
Single/divorced	150 (15.1)
Married	376 (39.1)
Widowed	462 (45.8)
**Domicile (n, (%))**	
Alone in private household	565 (56.1)
Living together with partner	375 (39.1)
Living with relatives/others	48 (4.8)
**GDS, mean (SD)**	1.81 (2.07)
Range	0–13
**EQ-5D VAS, mean (SD)**	73.52 (19.50)
Range	0–100
**LSNS, mean (SD)**	15.77 (5.93)
Range	0–30
**Number of illnesses**	3.58 (2.32)
Range	0–13

*Notes*. *SD* Standard deviation; *ISCED* International Standard Classification of Education; *GDS* Geriatric Depression Scale; *EQ-5D VAS* the visual analogue scale of the EQ-5D questionnaire; *LSNS* Lubben Social Network Scale

### Frequency and types of social loss experiences

**Fig. 2:**
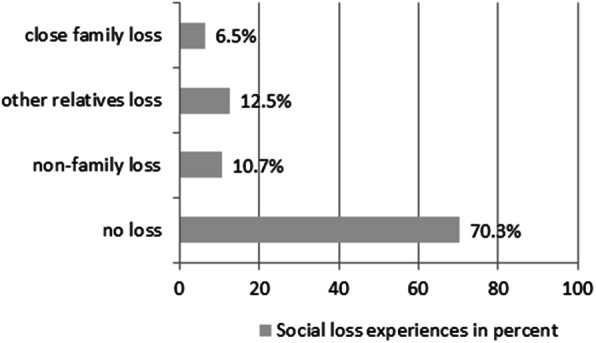
Frequencies and types of social loss experiences

Figure 2 shows social loss experiences during the previous 12 months. Out of 988 individuals aged 75 years and older, 291 (29.7%) experienced at least one social loss. Of those individuals, 63 (6.5%) experienced close family losses, 124 (12.5%) reported other relatives losses and 104 (10.7%) non-family losses.

### Social loss experiences and unmet needs

**Fig. 3:**
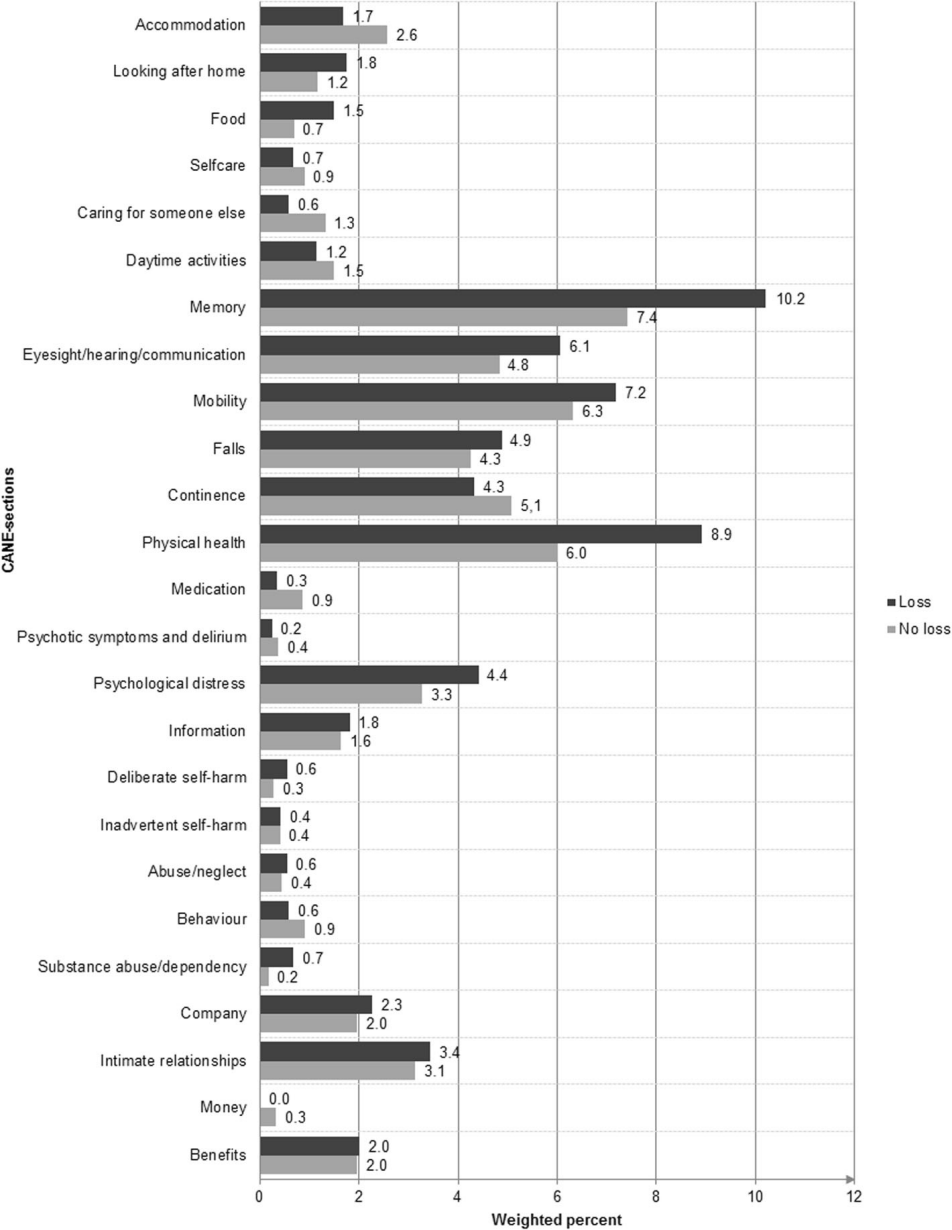
Unmet needs in elderly individuals with and without social loss experiences

Regarding different types of losses within the last 12 months, the highest proportion of individuals experiencing at least 1 unmet care need was found in individuals with close family losses (91.8%). This number was followed by individuals with non-family losses (71.8%), no losses (57.8%) and other relatives losses (48.2%). Figure 3 shows the frequencies of unmet needs for individuals with and without loss experiences during the past 12 months. Overall, individuals with loss experiences reported more unmet needs across all CANE sections than individuals without loss experiences. However, these differences were only observed on a descriptive level and did not reach statistical significance according to chi-square tests. Regardless, most unmet needs were reported in the CANE sections memory, followed by physical health, mobility, eyesight/hearing/communication, and falls. Accordingly, psychological and physical unmet needs played the most important role in a sample of individuals aged 75 years and older.

**Fig. 4:**
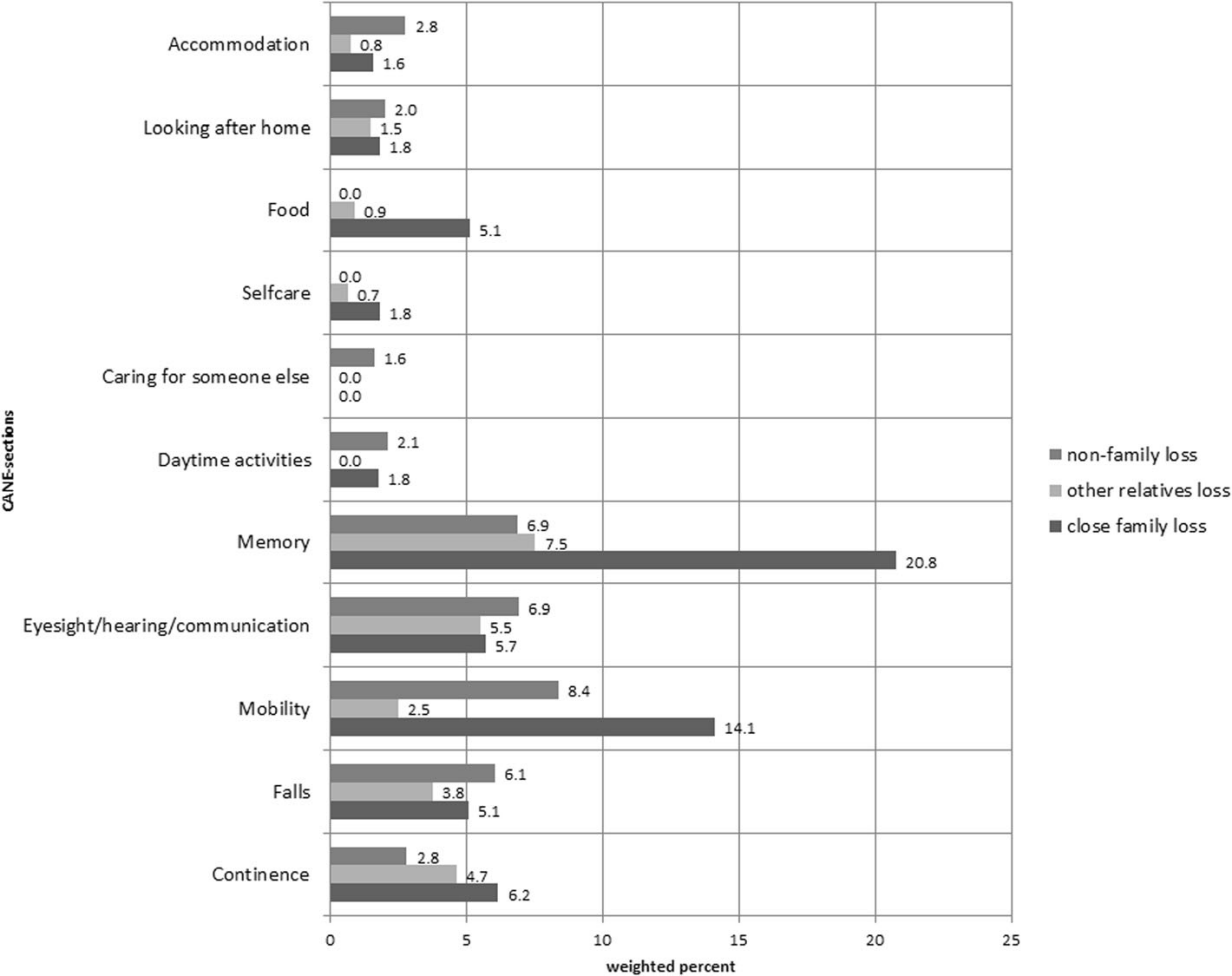
Unmet needs in elderly individuals with regard to different types of losses

**Fig. 5:**
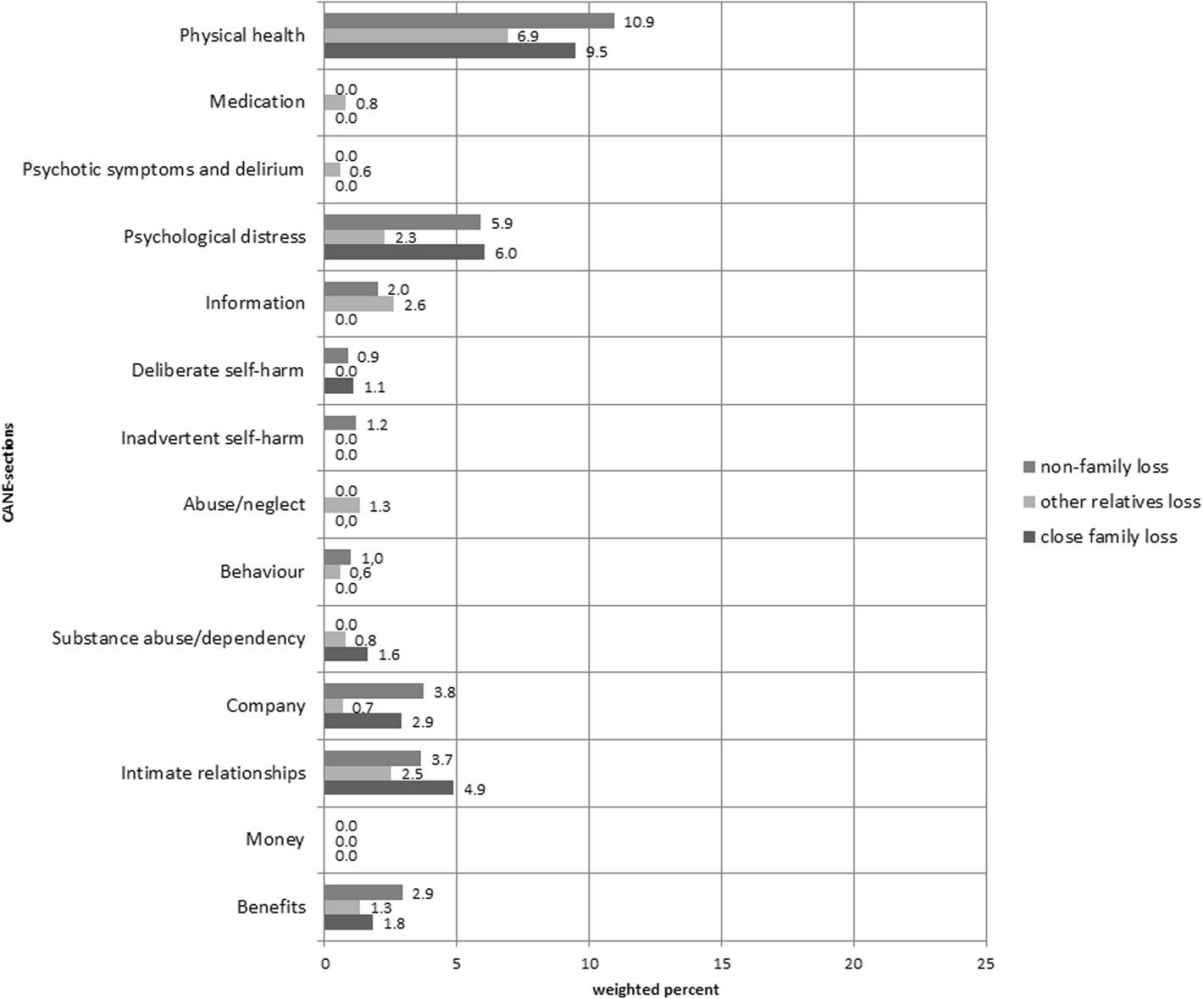
Unmet needs in elderly individuals with regard to different types of losses (continuation)

In addition, Figs. 4 and 5 are giving an overview of the frequencies of unmet needs in individuals with different types of losses. Elderly people who experienced close family losses within the last 12 months showed the highest proportions of unmet needs with regard to the CANE sections memory (20.8%) and mobility (14.1%).

In Table 2, the results of the negative binomial regression analyses for the prediction of unmet needs are shown. First, a significant association between close family losses with unmet needs was found. The incidence rate of unmet needs for individuals who experienced a close family loss during the past 12 months was 1.86 (95% CI 1.28–2.69) times the incidence rate for individuals who experienced no loss, holding all other factors constant. In other words, the number of unmet needs was 86% higher in those who experienced a close family loss. In addition, associations for age, marital status, depressive symptoms (GDS), social network (LSNS) and number of illnesses were found. Older patients were more likely to report unmet needs; each year of age increased the percentage of unmet needs by approximately 3%. Widowed individuals had a (1–0.57) × 100 = 43% reduction in the number of unmet needs compared to singles and divorced individuals. Depressive symptoms were associated with a higher number of reported unmet needs (IRR 1.23, 95% CI 1.17–1.29), whereas a higher social engagement was associated with a lower number of reported unmet needs (IRR 0.97, 95% CI 0.95–0.99). Finally, a higher number of illnesses indicating more severe morbidity of individuals significantly increased the number of unmet needs by the factor 1.09 (95% CI 1.04–1.15).

**Table 2:** Results of the negative binomial regression analysis for cross-sectional prediction of unmet needs

Model-Variables	IRR	RSE	z	*p*-value	95% CI
Social loss experiences (within last 12 months)^a^	Chi2 (3) = 13.15, *p* = .004				
* Close family loss (partner/child)*	1.857	0.352	3.268	**.001**	1.281–2.691
* Other relatives loss (siblings/other relatives)*	0.938	0.178	−0.339	.735	0.647–1.360
* Non-family loss (friends/neighbors/others)*	1.363	0.251	1.678	.093	0.949–1.957
Female	1.040	0.147	0.279	.780	0.789–1.372
Age	1.026	0.013	2.055	**.040**	1.001–1.051
Marital Status^b^	Chi2 (2) = 15.26, *p* = .001				
* Married*	0.812	0.292	−0.579	.562	0.402–1.642
* Widowed*	0.569	0.083	−3.862	**<.000**	0.427–0.758
ISCED^c^	Chi2 (2) = 1.36, *p* = .506				
* Moderate*	1.107	0.212	0.531	.595	0.761–1.611
* High*	1.226	0.236	1.061	.289	0.841–1.788
Domicile^d^	Chi2 (2) = 3.44, *p* = .179				
* Living together with partner*	0.740	0.261	−0.853	.394	0.370–1.479
* Living with relatives/others*	1.471	0.358	1.584	.113	0.913–2.371
GDS	1.228	0.033	7.745	**<.000**	1.166–1.294
EQ-5D VAS	0.998	0.003	−0.492	.623	0.992–1.005
LSNS	0.969	0.010	−2.902	**.004**	0.949–0.990
Number of illnesses	1.091	0.028	3.421	**.001**	1.038–1.146
Constant	0.066	0.075	−2.390	.017	0.007–.613
lnalpha	−.1509662	.1775947			−0.499 – 0.197
Alpha	.8598767	.1527096			0.607–1.218

*Notes. IRR* Incidence Rate Ratio; *RSE* Robust Standard Error; *z* test statistic; *CI* Confidence Intervall; ^a^Reference group for social loss experiences = no loss; ^b^Reference group for marital status = single/divorced; ^c^ISCED = International Standard Classification of Education, reference group = low; ^d^Reference group for domicile = living alone in private household; *GDS* Geriatric Depression Scale; *EQ-5D VAS* the visual analogue scale of the EQ-5D questionnaire; *LSNS* Lubben Social Network Scale

## Discussion

The current study delivers data on loss experiences and unmet needs among the representative German population aged 75 years and older. Furthermore, this study provides data on the association between loss experiences, socio-demographic and clinical factors and the frequency of unmet needs. As a main result, our study showed that loss experiences, especially those of a close family member, play an important role with regard to negative health outcomes such as unmet care needs.

Our results on the frequency of loss experiences (29.7%) were comparable to the results of a previous study [33] that found that 23% of participants reported a recent loss. However, comparison of results is possible only to a limited extent because these authors only focused on nonspousal loss experiences in a much younger sample compared to our study (65+ vs. 75+ years). The current study, allowed a broader assessment of social loss experiences taking into account various categories of lost loved ones including close family losses, other relatives losses and non-family losses. This represents a substantial extension compared to previous research mainly focusing on spousal loss experiences and effects of widowhood on health and psychological outcomes [3, 4, 34–36].

Our data showed that approximately 50% of the sample aged 75+ years and older was widowed at the time of the baseline assessment. This is in line with findings that widowed individuals appear to be older and the proportion of widows and widowers raises with increasing age [33, 37]. Further, the incidence rates of widowhood become less frequent in very old age [38]. Correspondingly, close family losses (including spousal loss) occurred less often in our sample and the most frequently reported loss experiences within the chosen time frame of the last 12 months were other relative losses, followed by non-family losses. While there is great consensus in scientific literature that loss experiences represent negative life events in older adults with large negative impact on health outcomes [5], there is almost no knowledge about the association of loss experience and unmet care needs in the oldest old. Thus, our data confirm earlier findings that loss experiences are associated with negative health outcomes such as lower life satisfaction [7], depressive symptoms and decreased functional status [38]. In addition, our study results show that loss experience is also associated with increased psychological and physical unmet care needs in this population.

In fact, the present study findings show that close family losses had the most relevant impact on unmet needs as compared to other relative losses and non-family losses. This finding does not seem surprising behind the backdrop that spouses or children often assist with a great number of daily acitivities, such as personal care or household tasks [12, 39]. Nevertheless, also elderly people with non-family losses showed unmet needs in a variety of categories. This finding is underlined by the results of a Dutch representative survey [40]. Accordingly, informal caregivers such as friends or neighbors play an important role in providing care for elderly people [40]. Consequently, not only close family losses, but also non-family losses may strongly affect elderly people with regard to unmet care needs. Clinicians such as general practitioners should take this into account when evaluating the care situation of an elderly person in their daily practice.

Other authors reported that loss experiences were linked to increased mortality [5]. Moreover, Jin and Chrisatakis showed that the link between increased mortality risk after spousal loss is mediated by a decline in quality of health care [9]. Thus, the associations between loss experiences and negative health consequences seem to be very complex and the consideration of unmet health care needs after loss experiences is strongly recommended in late life.

It has been shown that health and psychological problems mostly occur in the first 12 months after a loss [5, 38]. In line with this, our study suggests that loss experiences are strongly associated with a higher number of unmet psychological and physical care needs after holding other factors constant. Our study also confirms previous results on risk factors for increased unmet needs including a higher age, marital status, depression, decreased social engagement and more severe morbidity [15, 25, 41–43]. Thus, the current and earlier findings highlight the importance of the implementation of tailored intervention programs targeting at high-risk older adults with recent loss experiences. The early and reliable detection of unmet needs via the CANE in this high-risk population of bereaved elderly may prevent serious risks or the development of physical and mental diseases. In this context, the association between loss experiences and unmet needs may have been affected by other variables that could not be taken into account in the current study, for example, personality factors or coping styles. Here, complex associations can be assumed and future studies should also consider such influencing factors.

### Strengths and limitations

Major strengths of our study refer to the population-representative database and the large sample size allowing transferability (generalizability) of study results to individuals aged 75 years and older in everyday health care conditions. To our knowledge, the current study was the first attempt to analyze the association between loss experiences and unmet needs in the oldest old population in Germany. Higher age groups represent an important target group, as both losses and the associated increased risk of unmet needs are of particular relevance. Need assessment was based on the adapted German version of the CANE, which represents an established method for the reliable and valid determination of met and unmet needs in the elderly [15, 16].

In this study, a telephone survey was conducted. In addition to economic aspects, an advantage of this method may has been that individuals may be more likely to talk about sensitive issues, problems, and associated unmet needs due to the relative anonymity of a phone call compared to a direct personal conversation. This could have counteracted the finding that in old age problems and complaints often remain undetected, tabooed or masked. As a result, prejudices or effects of social desirability, which are assumed to be increasingly manifested in face-to-face interactions, may have been reduced [44, 45].

However, this study is also subject to several limitations. The response rate of the telephone survey was only 42.3%. Therefore a potential recruitment bias may not completely be ruled out. With regard to the interview mode, possible distortionary effects cannot be completely ruled out. For example, telephone surveys may be associated with increased cognitive effort, and fatigue of respondents and interviewers as well as response tendencies could have occurred [45]. This circumstance was taken into account insofar as breaks were taken if necessary or the interview was divided and continued at another time. In order to counteract monotony and possible stereotypical answer patterns, the question order and length of the interview was created in a way to reduce respondent burden. Additionally, the interview was tested and adapted in a pretest before the start of the main survey. All interviewers received extensive training in order to ensure the reliability and validity of the information obtained through the telephone survey. Furthermore, the present study was based on cross-sectional data that do not allow causal statements. In addition, the current study refers to a broad time frame of “the last 12 months” for social loss experiences. Future studies should take more detailed information with regard to the time since loss into account.

## Conclusions

In conclusion, not only the loss of the spouse but also other loss experiences such as the loss of a friend or other relative were accompanied by negative consequences such as psychological and physical unmet needs. Loss experiences are common phenomena in old age with enhanced probability in the oldest old. The assessment of these specific unmet needs should be part of routine medical examination in order to support optimal health and social care in the elderly. Future research should expand on longitudinal study designs taking into account various factors that may have an impact on the relationship between loss experiences and unmet needs.

## Data Availability

The datasets generated and analyzed during the current study are not publicly available due ethical restrictions and patient confidentiality but are available from the corresponding author on reasonable request. Aggregated data are provided in the paper tables.

## Abbreviations

CANE: Camberwell Assessment of Need for the Elderly
CATI: Standardized structured computer-assisted telephone interview
ADM: Association of German Market and Social Research Agency
6-CIT: 6-item cognitive impairment test
LLL: Leipziger Lebensereignis-Liste
EQ-5D VAS: EuroQol visual analogue scale
LSNS-6: Lubben Social Network Scale
ISCED: International Standard Classification of Education
GDS: Geriatric Depression Scale
IRR: Incidence Rate Ratios
